# Supplementary figures and images for: Validation of a Compact and Self-Contained Pyrosequencing Platform for Clinical Screening of RAS Mutations in Thyroid Cancers
Source: Diagnostics (Basel). 2025 Feb 6;15(3):390. doi: 10.3390/diagnostics15030390 (PMC11817209; doi:10.3390/diagnostics15030390)

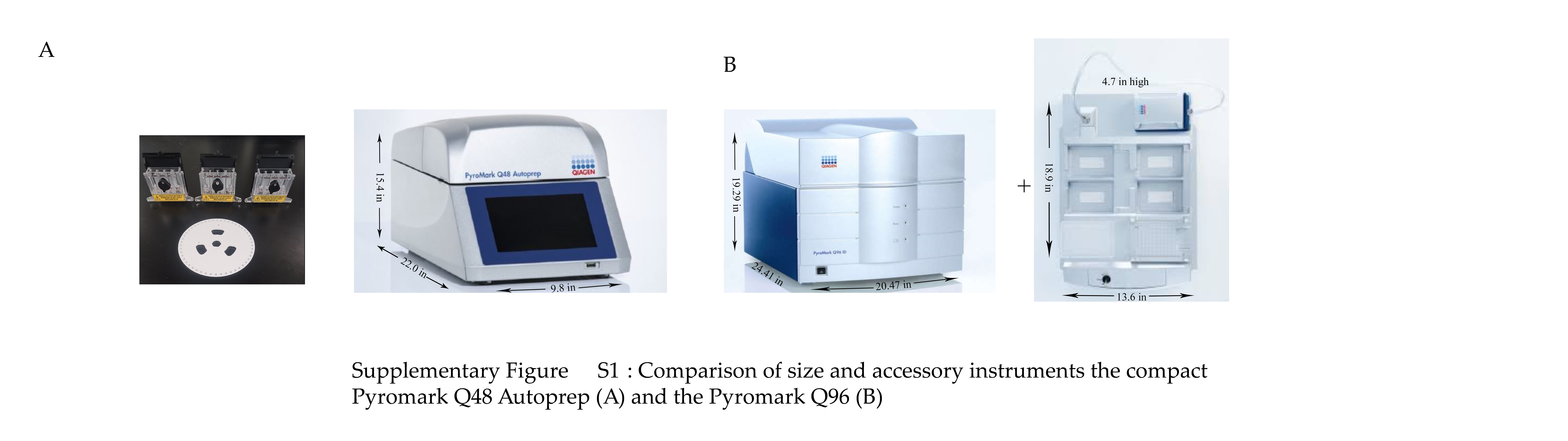

Supplement: Supplementary file 1 [file diagnostics-15-00390-s001.zip › Figure S1.tif]

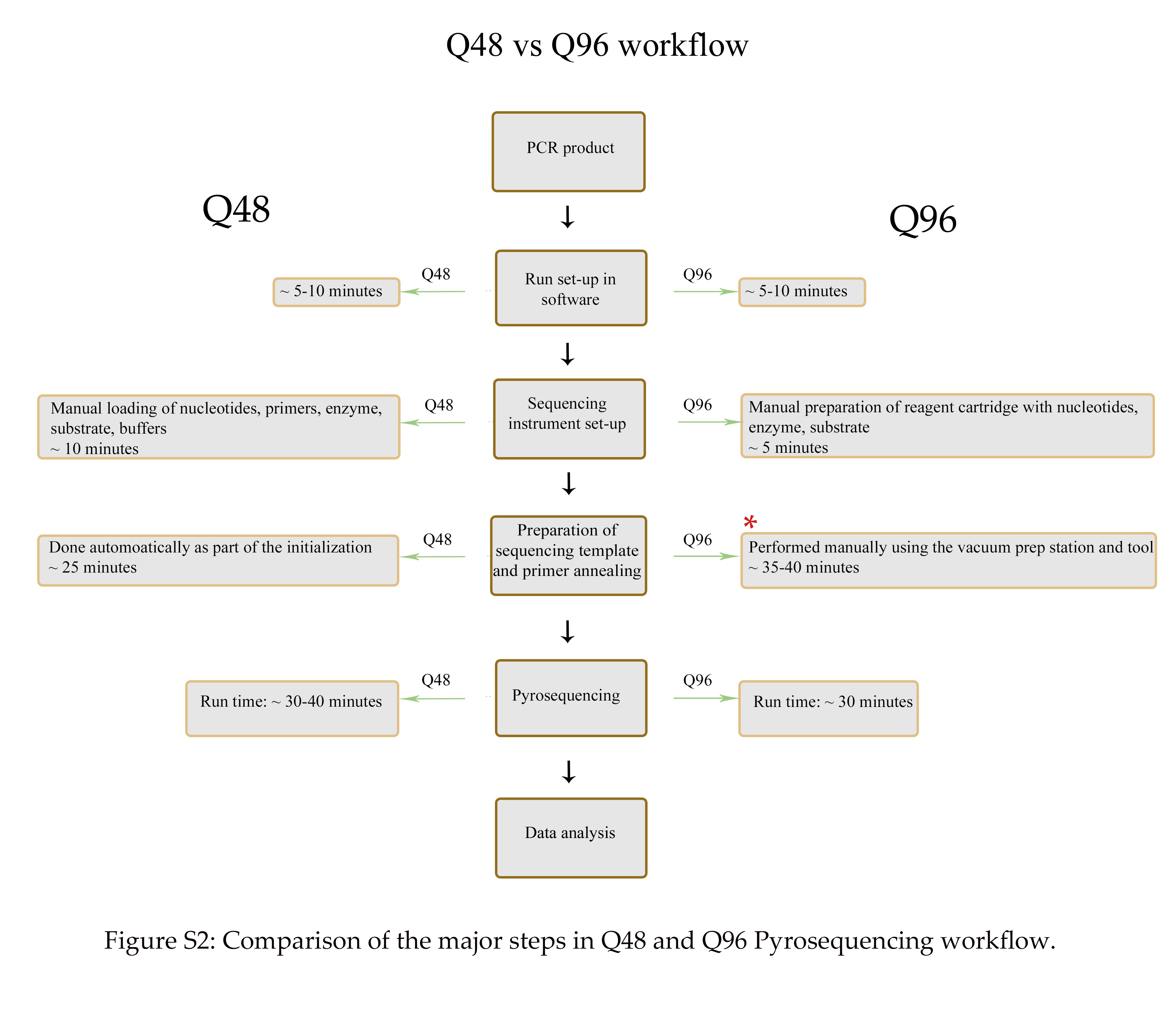

Supplement: Supplementary file 1 [file diagnostics-15-00390-s001.zip › Figure S2.tif]
